# Reduced muscle mass as predictor of intensive care unit hospitalization in COVID-19 patients

**DOI:** 10.1371/journal.pone.0253433

**Published:** 2021-06-17

**Authors:** Chiara Giraudo, Giovanni Librizzi, Giulia Fichera, Raffaella Motta, Elisabetta Balestro, Fiorella Calabrese, Giovanni Carretta, Anna Maria Cattelan, Paolo Navalesi, Michela Pelloso, Mario Plebani, Federico Rea, Roberto Vettor, Andrea Vianello, Roberto Stramare

**Affiliations:** 1 Department of Medicine–DIMED, University of Padova, Padova, Italy; 2 Respiratory Disease Unit, Department of Cardiac, Thoracic, Vascular Sciences and Public Health, University of Padova, Padova, Italy; 3 Pathological Anatomy Section, Department of Cardiac, Thoracic, Vascular Sciences and Public Health, University of Padova, Padova, Italy; 4 Unità Locale Socio Sanitaria–ULSS 3 Serenissima, Veneto Region, Italy; 5 Division of Infectious and Tropical Diseases, Padova University Hospital, Padova, Italy; 6 Anesthesiology and Intensive Care Unit, Department of Medicine–DIMED, University of Padova, Padova, Italy; 7 Department of Laboratory Medicine, University Hospital of Padova, Padova, Italy; 8 Thoracic Surgery, Department of Cardiac, Thoracic, Vascular Sciences and Public Health, University of Padova, Padova, Italy; 9 Internal Medicine, Department of Medicine—DIMED, University of Padova, Padova, Italy; 10 Respiratory Pathophysiology Division, Department of Cardiac, Thoracic, Vascular Sciences and Public Health, University of Padova, Padova, Italy; Universita degli Studi di Napoli Federico II, ITALY

## Abstract

**Purpose:**

To evaluate if reduced muscle mass, assessed with Computed Tomography (CT), is a predictor of intensive care unit (ICU) hospitalization in COVID-19 patients.

**Methods:**

In this Institution Review Board approved study, we retrospectively evaluated COVID-19 patients treated in our tertiary center from March to November 2020 who underwent an unenhanced chest CT scan within three weeks from hospitalization.We recorded the mean Hounsfield Unit (Hu) value of the right paravertebral muscle at the level of the 12^th^ thoracic vertebra, the hospitalization unit (ICU and COVID-19 wards), clinical symptoms, Barthel Index, and laboratory findings.Logistic regression analysis was applied to assess if muscle loss (Hu<30) is a predictor of ICU admission and outcome.Fisher’s exact and Student’s tests were applied to evaluate if differences between patients with and without muscle loss occurred (p<0.05).

**Results:**

One-hundred-fifty patients matched the inclusion criteria (46 females; mean age±SD 61.3±15 years-old), 36 treated in ICU. Patients in ICU showed significantly lower Hu values (29±24 vs 39.4±12, p = 0.001). Muscle loss was a predictor of ICU admission (p = 0.004).Patients with muscle loss were significantly older (73.4±10 vs 56.4±14 years), had lower Barthel Index scores (54.4±33 vs 85.1±26), red blood-cell count (3.9±1 vs 4.6±1*×10*^*12*^*L*^*−1*^), and Hb levels (11.5±2 vs 13.2±2*g/l*) as well as higher white blood-cell count (9.4±7 vs 7.2±4*×10*^*9*^*L*^*−1*^), C-reactive protein (71.5±71 vs 44±48*U/L*), and lactate dehydrogenase levels (335±163 vs 265.8±116*U/L*) (p<0.05, each).

**Conclusions:**

Muscle loss seems to be a predictor of ICU hospitalization in COVID-19 patients and radiologists reporting chest CT at admission should note this finding in their reports.

## Introduction

Muscle loss can be investigated by various radiological techniques including dual energy absorptiometry, magnetic resonance imaging (MRI), and computed tomography (CT) [1–6]. The latter which is overall the gold standard for the evaluation of body composition allowing a distinction of different tissues according to the attenuation of the X-ray beam, has been widely used for investigating and quantifying muscle loss not only in the elderly but also in patients who underwent prolonged hospitalization [7–11]. Moreover, in the last years, attention has been devoted to the socioeconomic impact of sarcopenia, as part of the so-called frailty syndrome, to the prognostic role of muscle loss in neoplastic patients as well as to its predictive function for postoperative infections [12, 13]. Regarding infectious diseases, recently, the impact of sarcopenia has started to be addressed also for COVID-19. In fact, researchers called for awareness raising about the occurrence of acute and chronic muscle loss in this group of patients and started to demand for tailored dietary and rehabilitation programs [14, 15].

Despite the utility of radiology for evaluating sarcopenia and the fact that worldwide the scientific community is looking for predictors of COVID-19 severity, to-date, imaging-based studies investigating the role of muscle composition in COVID-19 patients were still missing.

Thus, aim of our study was to evaluate if reduced muscle mass as defined at CT (i.e., <30 Hu) is a predictor of intensive care unit (ICU) hospitalization in COVID-19 patients [3, 4, 16].

## Material and methods

### Study design and muscle assessment

An electronic search of the database of our tertiary center was performed to identify patients with COVID-19 (i.e., positive at reverse transcription polymerase chain reaction) treated in our hospital from March 2020 to November 2020. The following inclusion criteria were then applied: i) adult COVID-19 patients (>18 years old) who underwent an unenhanced chest CT scan during the first three weeks of hospitalization; ii) chest CT scan including the paravertebral muscles at the level of the 12^th^ thoracic vertebra; iii) COVID-19 patients treated in intensive care unit (ICU) or COVID-19 wards.

The following exclusion criteria were established: i) patients who underwent a CT scan only after three weeks of hospitalization; ii) patients examined only with a contrast enhanced CT scan; iii) pediatric patients (<18 years old).

Given the inclusion/exclusion criteria, which do not apply any restrictions in terms of gender, ethnicity, and comorbidities, our sample can be considered representative of a larger population.

Patients or their guardians (i.e., for instance for sedated patients on mechanical ventilators) gave written informed consent to the CT scan as usually performed in clinical practice. Institution Review Board approval (i.e., Ethical Committee of Padova, n. 104n/AO/21) was obtained and written informed consent for participation to the study was waived due to the retrospective nature of the study.

**Fig 1:**
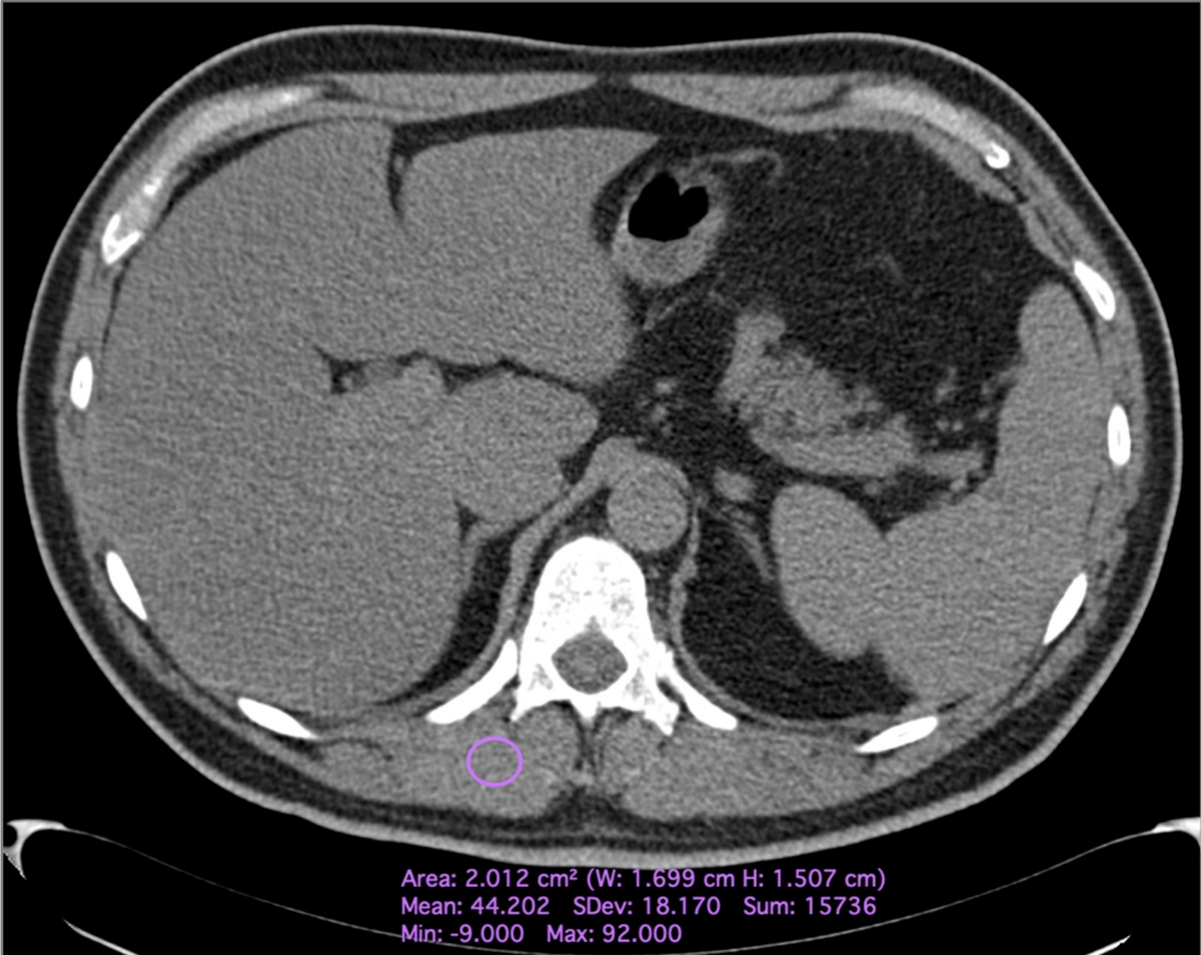
Axial Computed Tomography images of a 59 years-old male affected by COVID-19, hospitalized in a COVID-19 ward, demonstrating the applied method of measurements of muscle densitometry. A circular 2 cm size region of interest was placed on the right paravertebral muscle at the level of the 12^th^ dorsal vertebra and the mean Hounsfield unit value collected.

One radiologist with ten years of experience in musculoskeletal imaging collected the mean Hounsfield Unit (Hu) value of the right paravertebral muscle at the level of the 12^th^ thoracic vertebra using a standardized, circular region of interest (ROI) of 2 cm (Fig 1). All measurements were performed with an open source software (Horos v.3, www.horosproject.org).

Information regarding the unit of hospitalization considering ICU and COVID-19 wards, separately, was recorded. Moreover, we collected demographics (i.e., age and gender), Barthel Index (i.e., ordinal scale to measure the performance in activities of daily living; range 0–100) [17], and clinical symptoms at admission (i.e., fever >37.5° Celsius, any respiratory and gastrointestinal symptoms separately and any other symptom, such as neurological and cutaneous, grouped together) and laboratory findings on the same day of the CT (red (RBC) and white blood cell count (WBC), hemoglobin (Hb), C-reactive protein (CRP), creatine phosphokinase (CPK), aspartate aminotransferase (AST), alanine aminotransferase (ALT), alkaline phosphatase (ALP), and lactate dehydrogenase (LDH)).

### Statistical analyses

Descriptive statistics were applied for demographics. We used the binary logistic regression analysis for categorical independent variables to assess if reduced muscle mass, defined as Hu values <30, is a predictor of ICU admission and/or adverse outcome [3, 4, 16]. To further assess the accuracy of muscle Hu values in predicting the type of hospitalization (i.e., ICU or COVID-19 wards) we applied the receiver operating characteristic curve (ROC) and selected as cutoff the Hu value with the highest Youden index.

The student’s t-test was applied to assess if any difference regarding age, clinical, and laboratory findings occurred between patients with and without muscle loss while the Fisher’s exact test was used for the categorical variable gender.

To assess the robustness of the measurements, a second reader, with four years of experience in musculoskeletal imaging repeated the extraction of all HU values and the intraclass correlation coefficient (ICC), with average measures, was computed; values >.750 were considered as excellent [18].

All statistical analyses were performed using SPSS (IBM SPSS Statistics version 26, IBM Armonk, NY, USA) and applying p<0.05 as level of significance.

## Results

One-hundred-fifty patients were examined (46 females; mean age±SD 61.3±15 years old). The CT scans were performed on average 5.5±4 days after hospital admission. One-hundred fourteen patients were treated in COVID-19 wards only while 36 were hospitalized in ICU. Among all patients treated in ICU, nine died. Overall 43 patients (28.7%) were affected by reduced muscle mass (i.e., HU < 30), 16 of them hospitalized in ICU. One-hundred- thirty-six patients fully recovered while 14 deceased. Altogether six patients with muscle loss died and four of them were in ICU.

**Fig 2:**
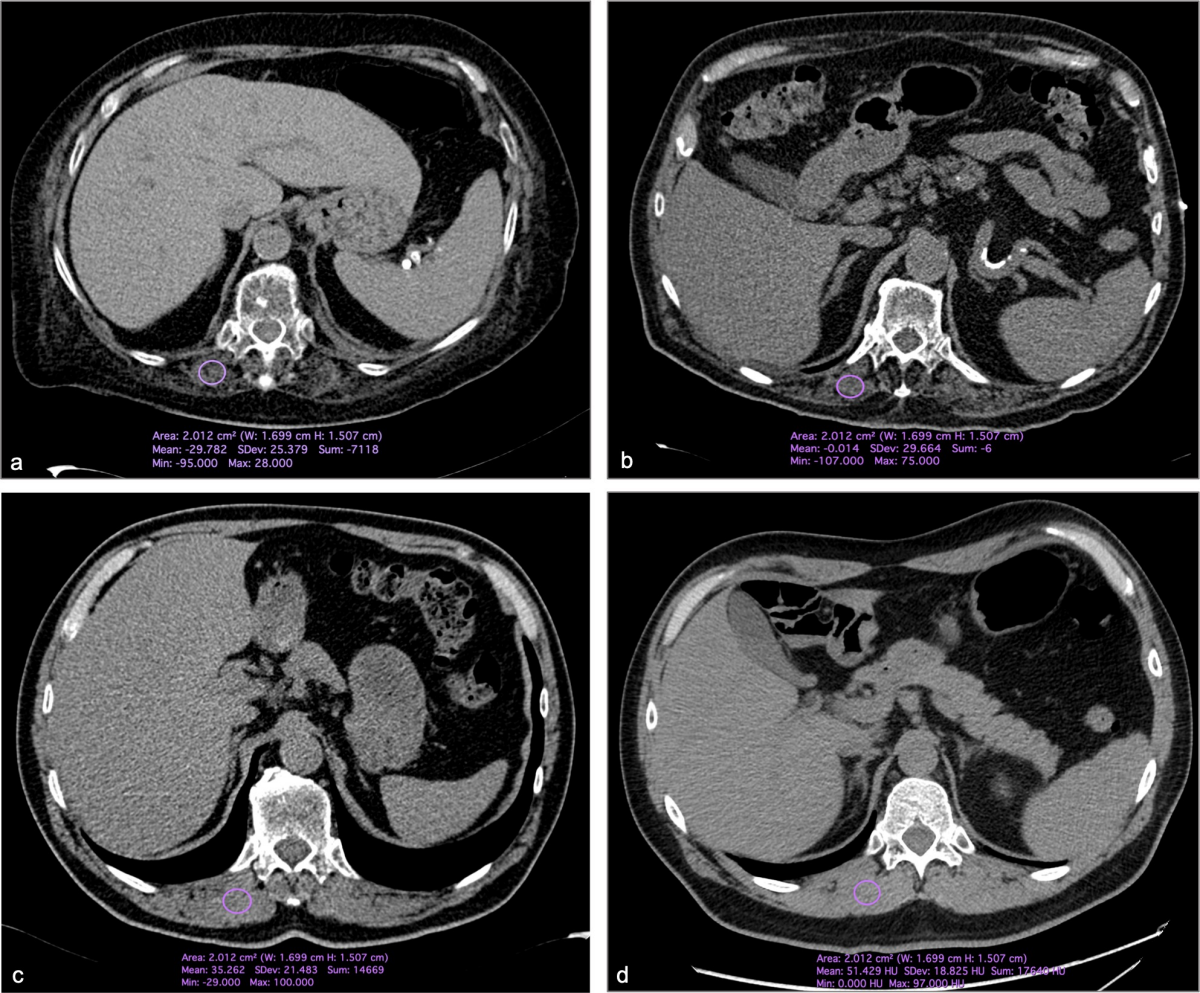
Axial Computed Tomography images of four patients with COVID-19. In particular, in a and b, one 74 years-old female and one 82 years-old male with reduced muscle mass (i.e., < 30 Hounsfield unit) hospitalized in intensive care unit. In c and d, two male patients (54 years old in a and 65 years old in b) hospitalized in COVID-19 wards demonstrating muscle values above the threshold for muscle loss.

Patients in ICU showed significantly lower Hu values (29±24 vs 39.4±12 Hu, p = 0.001) (Fig 2).

**Fig 3:**
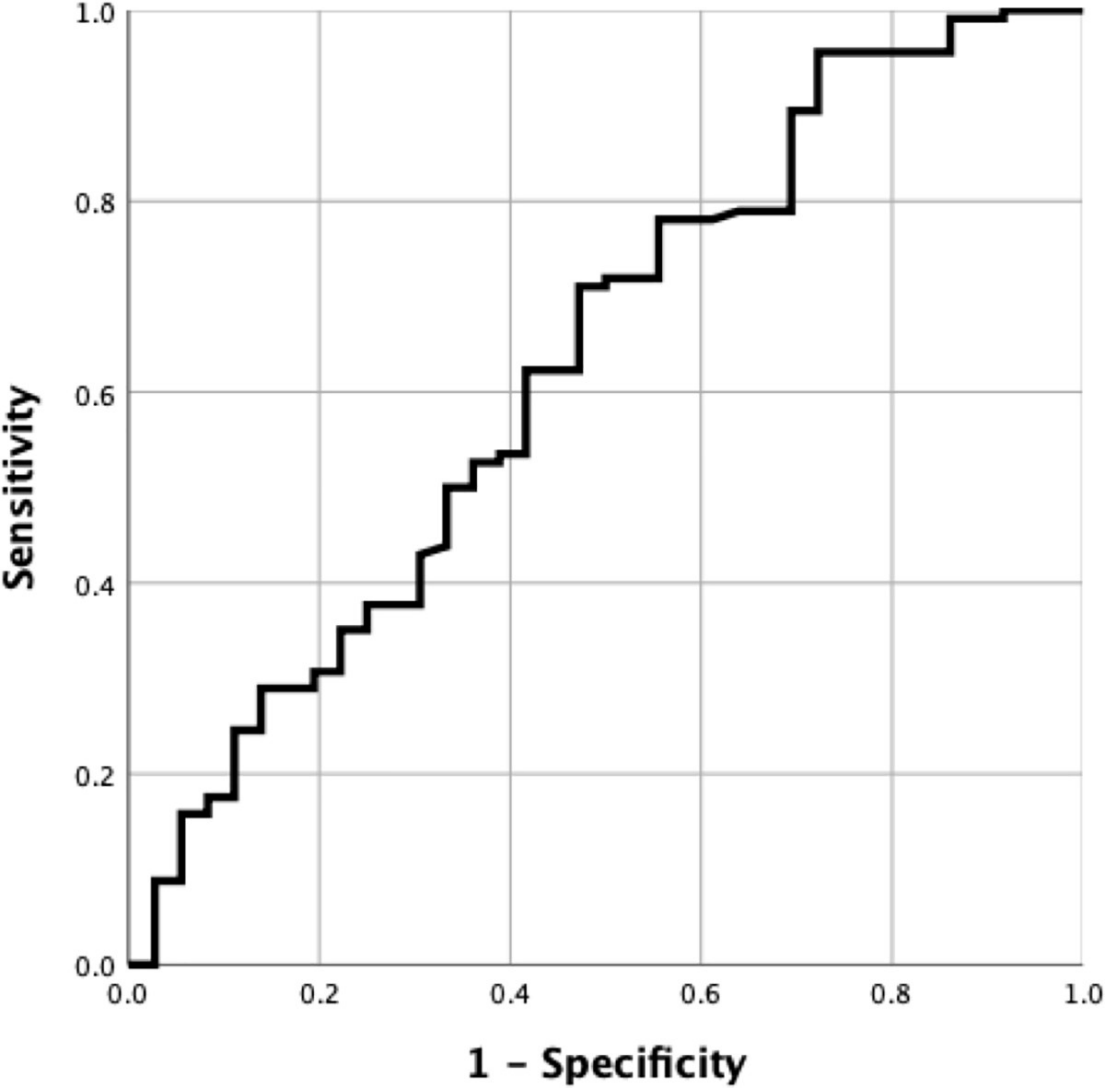
Muscle densitometry accuracy in classifying the unit of hospitalization. Receiver operating curve demonstrating the overall performance of muscle densitometry, expressed in Hounsfield unit, in classifying patients requiring hospitalization in intensive care unit (62.9% accuracy).

The logistic regression analysis showed that reduced muscle mass significantly influenced patients’ admission in ICU (p = 0.004). Moreover, the Hu values of muscles showed an overall accuracy of 62.9% in classifying the unit of hospitalization. In particular, a value of 34 Hu showed 71.1% sensitivity and 53% specificity for ICU admission (Fig 3). Muscle loss did not influence the overall outcome (p = 0.224).

The results of the comparison of demographics, clinical, functional independence, and laboratory findings between patients with and without reduced muscle mass are summarized in Table 1.

**Table 1:** Comparison of demographics, clinical, and laboratory findings between COVID-19 patients with and without muscle loss.

	*Patients with muscle loss*	*Patients without muscle loss*	*P*
*Gender*^*a*^ (female/male)	16/27	30/77	0.328
*Age (years)*^*b*^ (mean±SD)	73.4±10	56.4±14	**<0.001**
*Fever*^*a*^ (yes/no)	39/4	98/9	0.542
*Respiratory symptoms*^*a*^ (yes/no)	34/9	80/27	0.675
*Gastrointestinal symptomsr*^*a*^ (yes/no)	12/31	16/91	0.103
*Other symtpoms*^*a*^ (yes/no)	6/37	19/88	0.637
*Barthel Index* (mean±SD)	54.4±33	85.1±26	**<0.001**
*Hemoglobin (g/l)*^*b*^ (mean±SD)	11.5±2	13.2±2	**<0.001**
*Red blood cells count (×10*^*12*^*·L*^*−1*^*)*^*b*^ (mean±SD)	3.9±1	4.6±1	**<0.001**
*White blood cells count (×10*^*9*^*·L*^*−1*^*)*^*b*^ (mean±SD)	9.4±7	7.2±4	**0.019**
*C-reactive protein (mg/L*^*−1*^*)*^*b*^ (mean±SD)	71.5±71	44±48	**0.009**
*Aspartate aminotransferase (U/L)*^*b*^ (mean±SD)	62.1±139	40.7±39	0.154
*Alanine aminotransferase (U/L)*^*b*^ (mean±SD)	58.25±130	51.9±114	0.773
*Alkaline phosphatase (U/L)*^*b*^ (mean±SD)	79.7±37.8	69.3±38	0.195
*Lactate dehydrogenase (U/L)*^*b*^ (mean±SD)	335±163	265.8±116	**0.008**
*Creatine phosphokinase (U/L)*^*b*^ (mean±SD)	147±156	124.8±144	0.457

^a^Fisher’s exact test

^b^Student’s t-test; applied level of significance p<0.05.

In particular, patients with muscle loss were significantly older (73.4±10 vs 56.4±14 years old, p<0.001) had lower Barthel Index scores (54.4±33 vs 85.1±26, p<0.001), RBC (3.9±1 vs 4.6±1 10^^12^/L, p<0.001) and Hb levels (11.5±2 vs 13.2±2 g/L, p<0.001). Moreover, patients with reduced muscle mass showed significantly higher WBC (9.4±7 vs 7.2±4 10^^9^/L, p = 0.019), CRP (71.5±71 vs 44±48 mg/L, p = 0.009), and LDH values (335±163 vs 265.8±116 U/L, p = 0.008). No further statistically significant difference occurred between patients with and without reduced muscle mass for any other investigated variable (p>0.05, each).

The Hu measurements showed excellent reliability (ICC = .906, 95%CI .870 –.932).

## Discussion

This is the first radiological study assessing the role of reduced muscle mass in COVID-19 patients and demonstrating that it has an impact on ICU hospitalization. Although muscle loss did not carry a higher risk of mortality, our results are in line with previous research showing, for instance, that the skeletal muscle index is a predictor of progression in patients with sepsis and sarcopenia a predictor of postoperative infections and prolonged hospitalization in patients with colorectal cancer [13, 19–21]. Moreover, according to our data, the value of 34 Hu, even if slightly above the threshold for the definition of reduced muscle mass, indicates with high sensitivity which patients will be admitted to ICU. This finding suggests that even initial signs of muscle loss may have a significant impact on the severity and course of the disease. The underlying physio-pathological mechanism surely needs to be investigated also taking in account the role of laboratory analyses. In our cohort, the laboratory tests confirmed that patients with reduced muscle mass had a more severe disease (e.g., higher levels of LDH and CRP). Regarding in particular CPK, we found higher values in patients with low muscle mass albeit this difference was not significant. Similarly, an association between high levels of CPK and a worse prognosis and/or more severe disease has been recently described and several cases of COVID-19 induced rhabdomyolysis have been reported [22, 23]. Thus, not only should the potential onset of viral myositis due to the virus or the effect of immune mediated mechanisms be further investigated but also the role of angiotensin-converting enzyme receptors on muscles, which may contribute to the onset of myalgia and muscle loss [15, 22]. Certainly, it has to be underlined that in our cohort patients with reduced muscle mass were significantly older, confirming once more that the elderly are at high risk for a more severe course of the disease.

According to our evidence, during ICU hospitalization, despite all the challenges associated with this type of care, standardized physiotherapy programs based, for instance, on passive motion, rotational therapy, and stretching should be implemented and/or further promoted aiming to reduce the negative effect of prolonged immobilization especially in patients which may already be affected by muscle alteration at admission [24, 25]. Furthermore, longitudinal CT studies and/or research projects taking advantage of bedside techniques like ultrasound, which may guarantee a longitudinal monitoring of muscle loss avoiding all risks of contamination associated with patients’ mobilization/transfer to the radiology unit and reducing the radiation exposure, are expected to provide new insights into our findings [26–29].

Considering the overall social impact of the pandemic, Kirwan et al recently addressed the relationship between reduced muscle mass and COVID-19 on a broader level [30]. In fact, they highlighted that not only hospitalized patients may be affected by muscle loss but also the reduced physical activity due to quarantine, isolation and social distancing may cause a progressive loss of muscle tissue on healthy individuals and underlined the importance of home-based exercise programs [30].

This study is affected by several limits. First, information regarding the impact of prolonged hospitalization on muscles was not collected because it exceeded the aim of the study and during the first phase of the pandemic follow-up CTs were not performed. Further research should certainly be done in this direction to better tailor rehabilitation programs after hospital discharge and avoid/reduce the worse consequences of the frailty syndrome.

Recent evidence highlighted the role of several biochemical variables, like butyryl-cholinesterase, in the evaluation of sarcopenia but also because of the retrospective study design, we could not include such parameters [31]. Certainly, prospective studies on this topic should aim to a multivariate predicting model.

Muscle composition is usually assessed at the level of the third lumbar vertebra but most of COVID-19 patients undergo chest CT and such an approach would have not been feasible. Nevertheless, several studies already demonstrated that also using paravertebral muscles at the thoracic level provides reliable results [31–34].

Last, we did not perform a volumetric evaluation of paravertebral muscles’ composition, but we wanted to propose a method easily feasible in clinical practice. In fact, a circular region of interest can be easily drawn without any particular software of analysis and it is not time consuming. Thus, such an easily collectable information can be inserted in the clinical report also in an emergency setting aiming to provide beneficial information to clinicians and anesthesiologists dealing with COVID-19 patients. Moreover, the proposed approach has been already applied in the literature providing robust results as confirmed by our high reproducibility [3, 4, 9, 35–37].

## Conclusions

In conclusion, reduced muscle mass demonstrated to be a predictor of ICU admission in patients affected by COVID-19 and radiologists should not only become aware of this finding but, aiming to improve the quality of the delivered care, should embed this information in their report. We call for larger radiological studies, taking in account also the impact of prolonged hospitalization on the muscle tissue, to establish tailored rehabilitation programs after hospital discharge.

## Supporting information

S1 Data (XLSX)
